# Profiles of facial soft tissue changes during and after orthodontic treatment in female adults

**DOI:** 10.1186/s12903-022-02280-5

**Published:** 2022-06-26

**Authors:** Jie Gao, Xian Wang, Zaixiu Qin, Hao Zhang, Donghui Guo, Yuerong Xu, Zuolin Jin

**Affiliations:** 1 State Key Laboratory of Military Stomatology & National Clinical Research Center for Oral Diseases & Shaanxi Clinical Research Center for Oral Diseases, Department of Orthodontics, School of Stomatology, The Fourth Military Medical University, No. 169 Changle West Road, Xi’an, 710032 China; 2 Department of Stomatology, Haiyi Hospital, Zhoushan, 316000 China

**Keywords:** Orthodontic treatment, Facial soft tissue, 3dMD stereo photography, Extraction, Non-extraction, Facial aesthetics

## Abstract

**Background:**

Some female adults who received orthodontic treatment often complain about thinner faces, protruding cheekbones and sunken temples, even at the beginning of treatment. The present research aimed to explore facial soft tissue changes during and after orthodontic treatment, as well as the related factors affecting facial soft tissue changes.

**Methods:**

This study used 3dMD stereo photography technology to compare facial soft tissue changes among adult females who received orthodontics. A total of 52 adult females (24 teeth extraction, 28 non-teeth extraction cases) were included and potential correlations between related factors (facial morphology features, the change of occlusal height and dental arch width) were evaluated during different treatment periods.

**Results:**

Overall, 3D-negative soft tissue changes of the buccal region moderately correlated with distances of nasion-menton and subnasale-menton (both rs = 0.30, *P* < 0.05) as well as the ratio of subnasale-menton/right gonion-left gonion (rs = 0.33, *P* < 0.05) and nasion-menton/right zygomatic point-left zygomatic point (rs = 0.30, *P* < 0.05). Among the 3D angle measurements, the right chelion-median upper lip point-left chelion angle was found to have a moderate correlation with 3D negative changes of the upper cheilion region (rs = 0.31, *P* < 0.01). Analysis of occlusal height changes revealed that an increase in the posterior facial height (nasion-gonial distance) may be a risk factor for negative changes in the zygomatic arch area. In addition, a moderate positive correlation between the maxillary first molar width changes and 3D-negative changes of the lower cheek region was found (rs = 0.41, *P* < 0.05).

**Conclusions:**

After orthodontic treatment, adult females with wide and short faces may be prone to negative soft tissue changes. Changes of posterior facial height and arch width between the first molars were also risk factors for negative changes of facial soft tissues. Extraction is not a major factor producing facial soft tissue changes.

**Supplementary Information:**

The online version contains supplementary material available at 10.1186/s12903-022-02280-5.

## Background

In recent years, more and more adult patients, especially female patients, have received orthodontic treatment [1]. Adult patients usually seek treatment with clear goals, higher psychological requirements, and pay more attention to their appearance changes than younger patients [2]. In clinic work and social media some female adults who received orthodontic treatment often complain about thinner faces, protruding cheekbones and sunken temples, and they call this face change “braces face”. This even became a conflict between patients and physicians. However, it was found that the amount of bone change over a decade of aging is very limited [3] and during the process of 2–3 years of orthodontic treatment, the changes of facial shape mainly depend on changes in the soft tissue [4]. Conversely, there are few reports about the effect of adult orthodontic treatment on soft tissue, while it is not clear whether the new terminology "braces face" referring to thinner faces, protruding cheekbones and sunken temples is the result of excessive patient attention or the true effects of orthodontic treatments.

Cephalometric measurements of the cranio-maxillofacial region are used to develop orthodontic treatment plans and to evaluate orthodontic effects and treatment results [5, 6]. The traditional cranio-maxillofacial measurements mainly adopts a direct measurement approach, two-dimensional images and X-ray cephalometric measurements [7]. However, in modern orthodontics due to structural overlap and other reasons, two-dimensional images will lose some underlying information, and cannot display all the structures of facial soft tissues of patients. In recent years, with the development of 3D digital technology such as laser scanning, spiral CT or cone-beam CT computed tomography, and the wide application of 3D tomography in cranio-maxillofacial measurement, 3D digital technology has gradually replaced the traditional 2D technology [8] and has become an integral part of orthodontic applications. Due to its short shutter time and rapid image acquisition, stereo photography can significantly reduce the motion artifact of the image [9], which is convenient to record the soft tissue images of patients in clinical practice. Therefore, it has gradually received attention and favor from orthodontic clinicians in recent years.

The 3dMD face system (3dMD LLC, Atlanta, Ga) is a fast, high precision instrument designed for medical scientific investigations. It has a strong ability to adapt to light conditions by utilizing the actual surface information to create a spatial model. It can produce a 180° image of the face, from one ear to the other in about 1 ms. 3dMD can also measure the line distance, angle and volume of 3D images [10, 11]. The capture speed of 3dMD, no matter how many views, is about 1.5 ms or 1/650 s. The images captured during this short period can be considered as completely still [12].

In a previous study [9], the angles and distances between specific marks on faces of 10 adults were measured by direct anthropometry and findings compared with the results obtained by 3dMD. The results showed that there was no statistical differences in soft tissue measurement data between 3dMD and direct anthropometry [13], thus confirming the accuracy and precision of the 3dMD tomography system.

Whether the emergence of facial thinning associated with orthodontics is due to excessive attention of patients or the real effect of orthodontics has not been evaluated in western countries, probably due to the prominent features, high cheekbones and thin cheeks of Caucasian [14]. However, the faces of Asians are relatively flat [15], without prominent features.

The present study used 3dMD stereo photography technology to analyze trends of soft tissue changes in female adults after orthodontic treatments. The time course of development of any changes and whether the trend of soft tissue changes was consistent between extraction and non-extraction procedures were investigated. The factors associated with facial soft tissue changes were also explored.

## Methods

### Patients

From April 2014 to January 2017, data from 52 adult female patients (24 with teeth extractions, 28 without teeth extractions) who underwent orthodontic treatments in the Department of Orthodontics, Fourth Military Medical University were collected. The average patient age was 25.09 ± 4.04 years in the extraction group and 24.83 ± 3.46 years in the non-extraction group. Inclusion criteria were: (1) Chinese, Han nationality; (2) Aged 18–35 years; (3) Angle I or Angle II malocclusion, 6° > subspinale (A)-nasion (N)-supramental (B) [ANB] angle > 0°; (4) Patients who required or did not require dental extractions; (5) Initial body mass index (BMI) between 18.5 ≤ BMI < 24, and the fluctuation range of body weight at each 3dMD image collection was within the normal range; (6) Subjects voluntarily participated in the study and provided written informed consent. Exclusion criteria were: (1) Severe osseous asymmetry, open and closed deformity or partial deformity; (2) Maxillofacial trauma, surgery, orthodontic or a tooth extraction history; (3) Maxillofacial soft tissue development defects or soft tissue asymmetry more than 5 mm; (4) Temporomandibular joint disorders with obvious clinical symptoms.

All procedures followed the guidelines of the Declaration of Helsinki (2013) and given approval by the ethics committee of Hospital of Stomatology, Fourth Military Medical University (approval number IRB-REV-2021044). Written informed consent was obtained from all the patients.

### Orthodontic process

All patients were treated with a labial orthodontic system, with a bracket slot of 0.022 × 0.028 inches. For all tooth extraction cases, different anchorage requirements were selected according to the patient’s diagnosis and protrusion including microimplants or transpalatal arch (TPA) devices without any external anchorage devices. In all extraction patients, the time of tooth extraction was one week before the any bracket bonding. Four orthodontic teeth were extracted at one time. Only half of the brackets were affixed at the first time, and the bonding time of the lower half brackets was 1 month later. The second molars were treated in all cases at the later stage of orthodontic treatment.

### 3D measurements of facial morphology

**Fig. 1:**
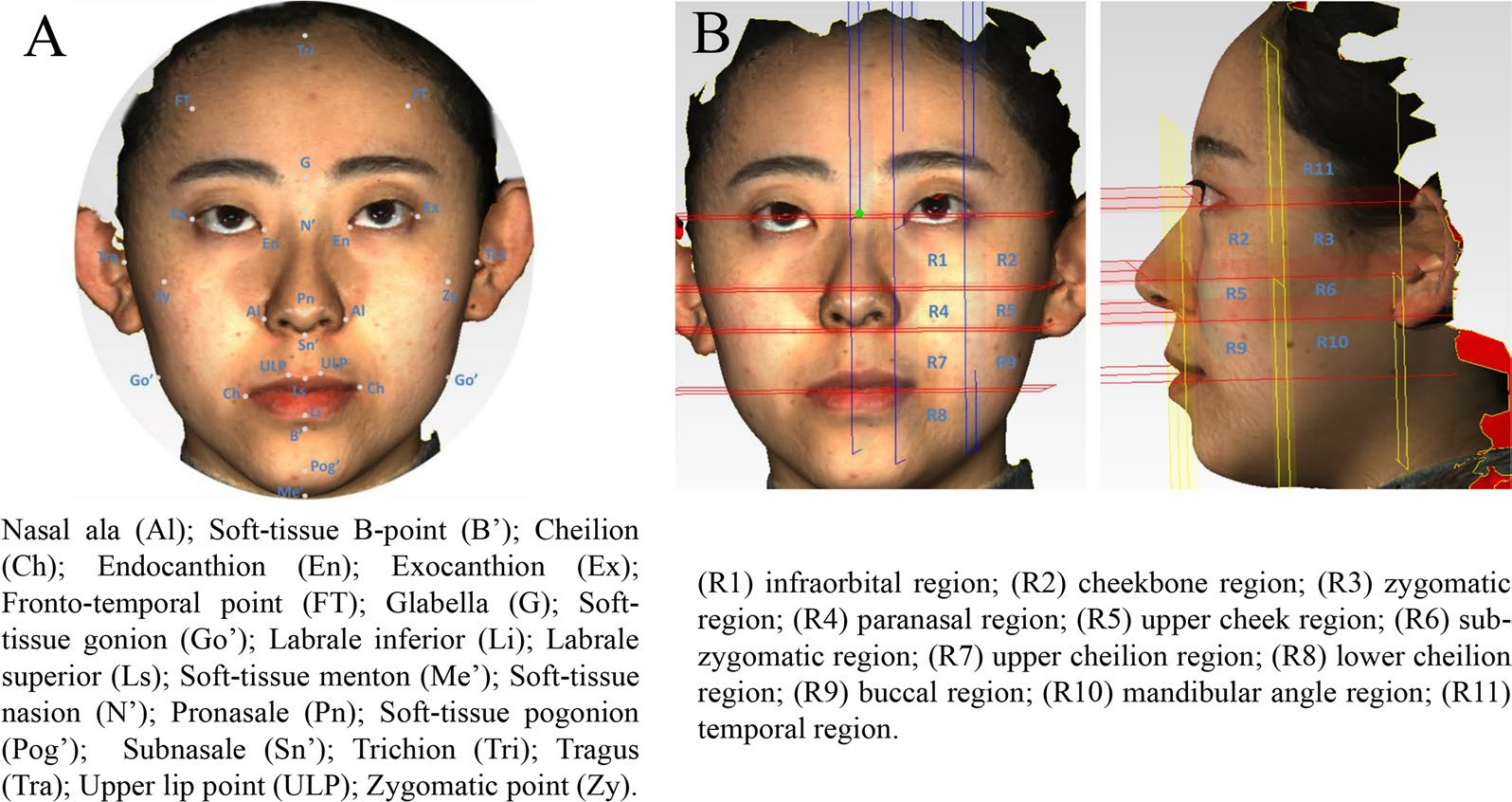
Illustrations of fix points for 3D measurements of the facial morphology. **A** soft tissue markers, **B** face regions for separate analysis

Facial 3D soft tissue features were measured before (T_0_) and after treatment (T_4_). Soft tissue markers are illustrated in Fig. 1A. 3D line distance, angles and ratios were measured according to the marker points. The angles of right (Rt) Soft-tissue gonion (Go')-soft-tissue pogonion (Pog')-left (Lt) Go' [RtGo'-Pog'-LtGo'], Rt cheilion (Ch)-upper lip point midline (ULPm)-LtCh [RtCh-ULPm-LtCh] and Rt zygomatic point (Zy)- pronasale (Pn)-LtZy [RtZy-Pn-LtZy] were measured after projection to the horizontal plane. The angles of soft-tissue nasion (N')-Pn⊥glabella (G)- Pog' [N'-Pn⊥G-Pog'], Lt tragus (Tra)-Lt nasal ala (Al)⊥LtGo'-soft-tissue menton (Me') [LtTra-LtAl⊥LtGo'-Me'], and [RtTra-RtAl⊥RtGo'-Me' were measured after projection to the sagittal plane [15–19].

### Division of measurement areas and image measurements

Four horizontal planes were determined according to the exocanthion, subnasal point, lateral tragus point and cheilion. Four sagittal planes were identified according to the endocanthion and exocanthion on both sides. Three coronal planes were determined according to the subnasal point, bilateral lateral tragus point, and the midpoint of the line between the lateral tragus point and the subnasal point. Then, the faces were divided into 11 regions relevant to this study (Fig. 1B). In this manner, the face can be divided into 11 regions on one side and 22 regions on both sides, which will not only illustrate the soft tissue changes comprehensively, but also focus on the changes in each region.

### 3D data collection

The 3D facial data of the orthodontic patients were collected before bracket bonding (T_0_), 3 months after treatment initiation (T_1_), 6 months after treatment initiation (T_2_), 12 months after treatment initiation (T_3_), and at the end of treatment after bracket removal (T_4_). T_0_ and T_1_–T_4_ images were imported into Geomagic Qualify software (Geomagic 2013, Research Triangle Park, NC) to generate a 3D head model with high accuracy and color information. The obtained 3D data at T_1_, T_2_, T_3_ and T_4_ were overlapped with the images at T_0_ to calculate the facial changes at different time periods. A detailed description of face change data acquisitions is provided as Additional File 1: Fig. S1 and S2 [15].

### Measurement of occlusal height change

#### Image measurement

**Fig. 2:**
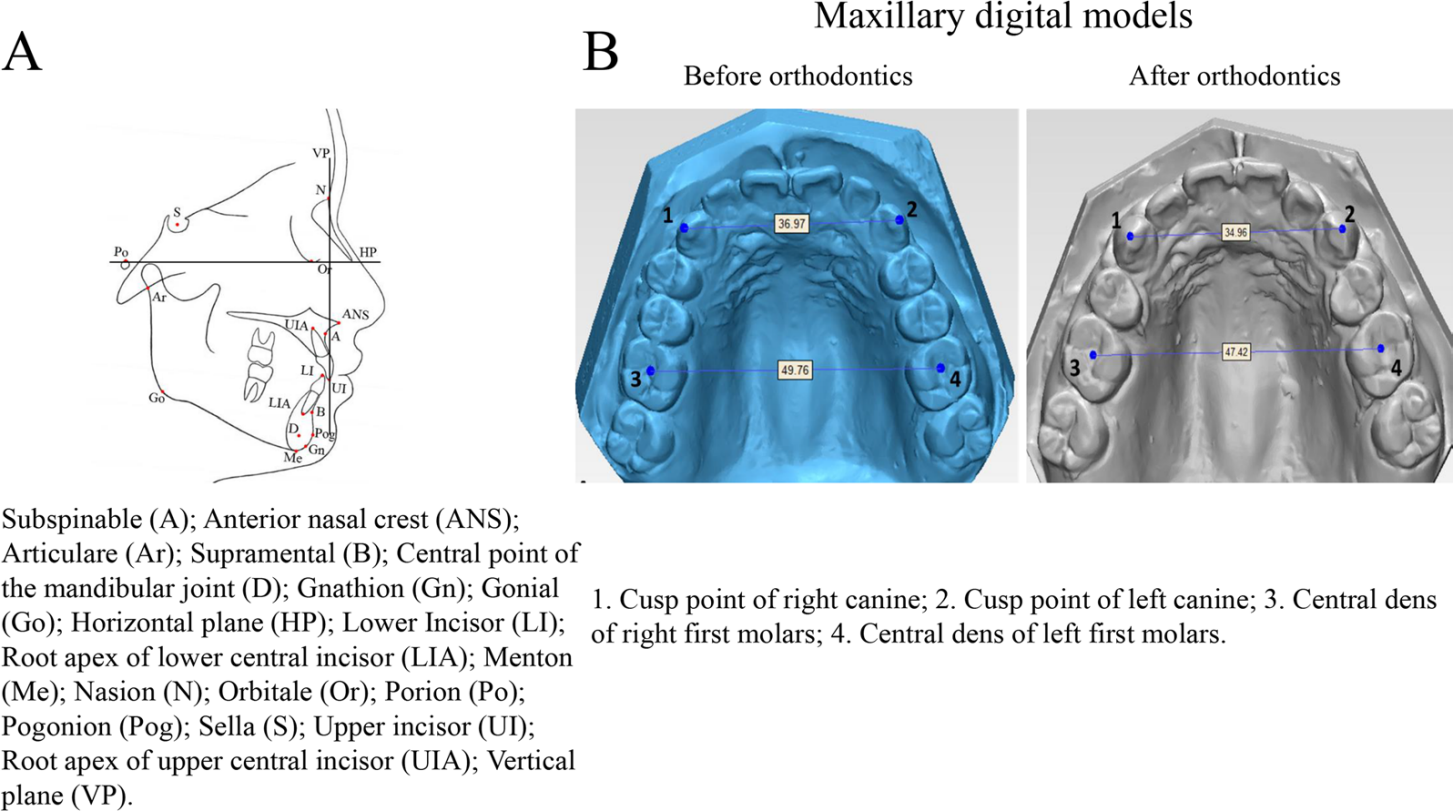
Cephalometric and digital maxillary model illustrations. **A** cephalometric marker points, **B** marker points and measurement items of the maxillary digital model

Cephalometric lateral radiographs (T_0_ and T_4_) were imported into Onyx 2.6 in BMP format (Image Instruments, Chemnitz, Germany) and the brightness and contrast were adjusted to make the soft and hard tissues clearly visible. Some measurement items in Steiner analysis [20], Tweed analysis [21] and Coben analysis [22] were selected for cephalometric analysis. Mark points used in X-ray cephalometric analysis are shown in Fig. 2A. The orbital point (Or) and Porion point (Po) were connected for constructing the Frankfort horizontal plane (HP), from which a line perpendicular to the Frankfort horizontal (FH) plane was constructed through the nasion (N) as the vertical plane (VP) (Fig. 2A). The measurement items used in cephalometric analysis were: 1. Sella (S)-NA [SNA]; 2. SNB; 3. ANB; 4. SN-D-point (D) [SND]; 5. Upper incisor (UI)-NA [UI-NA] angle; 6. UI-NA distance; 7. Lower incisor (LI)-NB [LI-NB] angle; 8. LI-NB distance; 9. UI-LI angle; 10. Gonial (Go) menton (Me)-LI [GoMe-LI] angle; 11. N-Me (Anterior facial height); 12. Anterior nasal crest (ANS)-Me [ANS-Me] (Anterior lower facial height); 13. N-Go (Posterior facial height); 14. Articulare (Ar)-Go (Posterior lower facial height) and 15. GoMe- HP plane angle [23–26].

### Measurement of dental arch width

#### Model measurement

Dental plaster models were collected before and after orthodontic treatment from all subjects who met the inclusion criteria. The plaster model was scanned using 3Shape R700 3D digital model scanner (3Shape, Copenhagen, DNK) to obtain the digital model data of patients before and after treatment.

The maxillary digital model data before and after orthodontic treatment were imported into 3-Matic STL 9.0 software (Materialise, Leuven, BEL) in STL format for the determination of markers and model measurement. The markers on the maxillary model before and after correction are shown in Fig. 2B. Measurement items included the maxillary model before and after orthodontic treatment, maxillary canine width (the distance between the cusp points of left and right maxillary canine) and maxillary first molar width (distance between central fossa of left and right first molars). Measurement items were the distance between the cusp points of the left and right maxillary canine (1–2) and the distance between the central fossa of left and right first molars (3–4) (Fig. 2B) [27, 28].

### Statistical analysis

Point fixation and measurements were carried out once every week, three times in total, and the average was taken as the experimental result. SPSS ver. 22.0 software was used for descriptive statistics (mean ± SD) on BMI index, changes in the overall facial soft tissue area and changes in different facial soft tissue regions at different times. A paired *t*-test was used to compare the statistical differences between the left and right sides of the face in the same period (α = 0.05), to observe the facial and the trend of facial changes in female patients with or without tooth extraction at different times. A paired *t*-test was used for comparison of the initial ages between the non-extractive and extractive groups. The comparison of BMI at the same time, the general change of soft tissue and soft tissue change in different regions at the same time, were dependent on whether the variables were normally distributed. If the variables were normally distributed and the variances uniform, a *t*-test was used to analyze the differences between the non-extraction group and the extraction group, to determine whether there were differences in the overall facial changes, and in different regions during the same period of treatment. The obtained 3D facial morphology features (line distance, angle and ratio), occlusal height change, dental arch change and the 3D facial soft tissue change data of adult female were used for two-sided Spearman correlation analysis (α = 0.05). An error analysis description is provided as Additional file 1.

## Results

### Demographic and clinical characteristics of patients

There was no statistically significant difference in initial age between the extraction group and the non-extraction group (*P* = 0.800). As Table 1 shows, the initial and throughout different times BMI values of all included subjects were not statistically significantly different.

**Table 1:** Comparison of BMI in different periods

	Non-extraction	Extraction	*P-*value
T_0_	20.7 ± 1.55	20.1 ± 1.40	0.16
T_1_	20.7 ± 1.49	20.1 ± 1.26	0.16
T_2_	20.6 ± 1.50	20.3 ± 1.34	0.38
T_3_	20.7 ± 1.43	20.4 ± 1.32	0.32
T_4_	20.8 ± 1.56	20.4 ± 1.27	0.35

T_0_, before bracket bonding; T_1_, 3 months after treatment initiation; T_2_, 6 months after treatment initiation, T_3_, 12 months after treatment initiation; T_4_, at the end of treatment

### 3D morphology analysis in orthodontics groups before treatments

A comparison of 3D line distances is shown in Additional file 1: Table S1. There was no statistical difference in the facial 3D line distance between the extraction group and the non-extraction group before orthodontic treatment. The angle of subnasale (Sn')-labrale superior (Ls)⊥labrale inferior (Li)- soft-tissue B-point(B') [Sn'-Ls⊥Li-B'] of patients in the extraction group was about 14° smaller than that in the non-extraction group (*P* < 0.01). There was no statistical difference in other 3D angle measurement items (Additional file 1: Table S2). No statistical difference was found in the facial 3D ratio between the extractive group and the non-extractive group before orthodontics (Additional file 1: Table S3).

### Comparison of facial regions among the two groups at different time points during and after treatments

The soft tissue in 11 regions between extraction and non-extraction groups were compared at different periods (Table 2, Fig. 1B). The results showed that only the temporal area (R11) showed a statistical difference between two groups at T_1_–T_0_, which was − 1.76 mm in extraction group and − 1.36 mm in non-extraction group. No significant difference was found at T_2_–T_0_. While the changes at T_3_–T_0_ in R11 were − 1.44 mm and − 1.10 mm for extraction and non-extraction groups, respectively, showing a remarkable difference. Similarly, at T_4_–T_0_ period, only the changes in R11 were significant (− 1.43 mm in extraction group vs. − 1.05 mm in the non-extraction group). Overall, the results showed that there was no difference of variation trend in different facial regions in patients with or without tooth extraction. The facial changes of extractive and non-extractive patients were consistent, which again indicates that extraction is not the main factor influencing facial changes of adult orthodontic patients. For non-extraction patients, the same changes can occur as well.

**Table 2:** Comparison of facial regions at different periods ($\overline{\mathrm{X} }$**±SD**)

Region	Non-extraction	Extraction	*P*-value	Non-extraction	Extraction	*P*-value	Non-extraction	Extraction	*P*-value	Non-extraction	Extraction	*P*-value
	T_1_–T_0_	T_1_–T_0_		T_2_–T_0_	T_2_–T_0_		T_3_–T_0_	T_3_–T_0_		T_4_–T_0_	T_4_–T_0_	
W	− 2.59 ± 0.35	− 2.56 ± 0.31	0.768	− *2.4* ± 0.39	− 2.76 ± 0.62	*0.068*	− *2.21* ± 0.42	− *2.54* ± 0.50	*0.101*	− *2.13* ± 0.41	− *2.54* ± 0.37	*0.657*
R1	− 1.27 ± 0.52	− 1.21 ± 0.40	0.717	− *1.11* ± 0.41	− *0.93* ± 0.37	*0.099*	− *0.93* ± 0.36	− *1.00* ± 0.44	*0.577*	− *0.98* ± 0.30	− *0.98* ± 0.32	*0.938*
R2	− 1.01 ± 0.54	− 1.18 ± 0.39	0.245	− 0.88 ± 0.39	− 1.01 ± 0.40	0.271	− 0.86 ± 0.39	− 0.89 ± 0.34	0.779	− 0.93 ± 0.54	− 0.85 ± 0.37	0.507
R3	− 1.07 ± 0.43	− 1.05 ± 0.79	0.857	− 1.05 ± 0.40	− 1.07 ± 0.35	0.88	− 0.74 ± 0.30	− 0.94 ± 0.39	0.068	− 0.93 ± 0.30	− 0.91 ± 0.34	0.814
R4	− 0.65 ± 0.3	− 0.73 ± 0.38	0.474	− 0.65 ± 0.38	− 0.72 ± 0.43	0.557	− 0.70 ± 0.35	− 0.91 ± 0.40	0.317	− 0.85 ± 0.33	− 0.89 ± 0.35	0.691
R5	− 0.89 ± 0.48	− 1.04 ± 0.84	0.455	− 0.71 ± 0.38	− 1.15 ± 0.36	0.317	− 0.60 ± 0.29	− 0.77 ± 0.37	0.061	− 0.84 ± 0.32	− 0.97 ± 0.43	0.257
R6	− 1.08 ± 0.50	− 1.18 ± 0.51	0.5	− 0.88 ± 0.48	− 1.22 ± 0.43	0.081	− 0.76 ± 0.23	− 1.13 ± 0.32	0.242	− 0.97 ± 0.35	− 1.06 ± 0.38	0.401
R7	− 0.84 ± 0.42	− 1.02 ± 0.43	0.171	− 0.80 ± 0.47	− 1.09 ± 0.54	0.052	− 0.83 ± 0.38	− 1.19 ± 0.33	0.064	− 1.07 ± 0.24	− 1.03 ± 0.45	0.71
R8	− 1.2 ± 0.66	− 1.49 ± 0.66	0.152	− 1.25 ± 0.55	− 1.87 ± 0.59	0.254	− 1.21 ± 0.48	− 1.62 ± 0.56	0.439	− 1.18 ± 0.38	− 1.55 ± 0.59	0.062
R9	− 1.16 ± 0.5	− 1.37 ± 0.81	0.293	− 1.05 ± 0.42	− 1.31 ± 0.32	0.252	− 0.92 ± 0.39	− 1.15 ± 0.31	0.144	− 1.05 ± 0.29	− 1.23 ± 0.39	0.067
R10	− 1.17 ± 0.5	− 1.46 ± 0.52	0.27	− 1.04 ± 0.47	− 1.26 ± 0.42	0.084	− 0.81 ± 0.28	− 1.31 ± 0.36	0.113	− 1.03 ± 0.28	− 1.11 ± 0.40	0.422
R11	− 1.36 ± 0.48	− 1.76 ± 0.50	0.008**	− 1.35 ± 0.56	− 1.59 ± 0.47	0.115	− 1.10 ± 0.40	− 1.44 ± 0.41	0.005**	− 1.05 ± 0.33	− 1.43 ± 0.49	0.002**

R1, infraorbital region; R2, cheekbone region; R3, zygomatic region; R4, paranasal region; R5, upper cheek region; R6, sub-zygomatic region; R7, upper cheilion region; R8, lower cheilion region; R9, buccal region; R10, mandibular angle region; R11, temporal region; T_0_, before bracket bonding; T_1_, 3 months after treatment initiation; T_2_, 6 months after treatment initiation, T_3_, 12 months after treatment initiation; T_4_, at the end of treatment; W, Overall facial area. ** *P* < 0.01

Values are all expressed as mean ± SD. *Significant difference between the Non-extraction and Extraction groups with *P* < 0.05; **Significant difference between the Non-extraction and Extraction groups with *P* < 0.01

### Analysis of related factors affecting facial soft tissue following orthodontics

#### Correlation analysis of adult female facial morphology and 3D facial soft tissue changes after orthodontic treatment

**Fig. 3:**
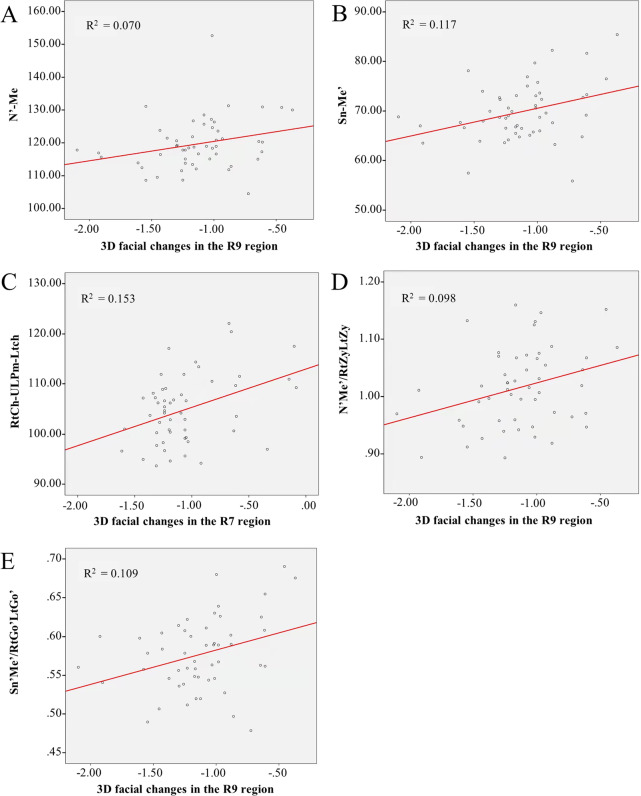
Correlation analysis of adult female facial morphology and 3D facial soft tissue changes after orthodontic treatment. **A** Correlation between the R9 and N'-Me' distance; **B** Correlation between the R9 and Sn'-Me' distance; **C** Correlation between the R7 and RtCh-ULPm-LtCh angle; **D** Correlation between the R9 and N'-Me'/RtZy-LtZy ratio; **E** Correlation between the R9 and Sn'-Me'/RtGo'-LtGo' ratio. Ch, cheilion; Go', soft-tissue gonion; Lt, left; Me', soft-tissue menton; N', soft-tissue nasion; R7, upper cheilion region; R9, buccal region; Sn', subnasale; ULPm, upper lip point midline; Zy, zygomatic point

**Fig. 4:**
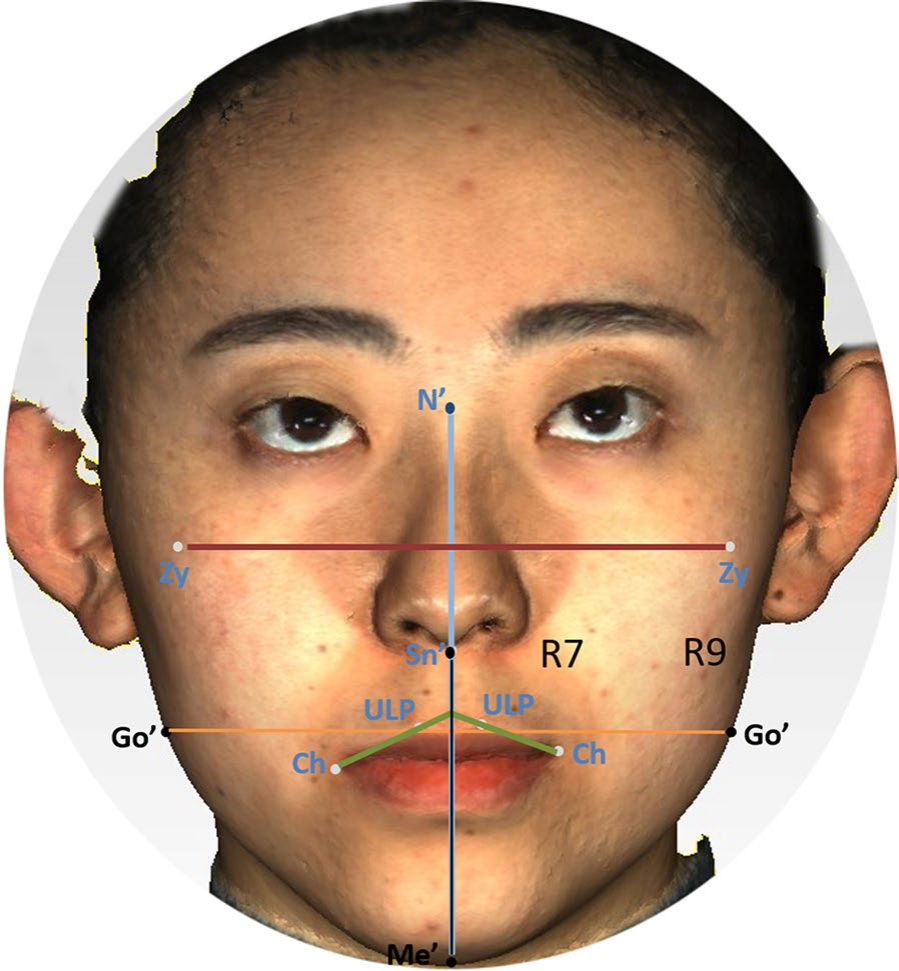
Scheme of soft tissue change parameters between baseline and T_4_ after orthodontic treatments. Ch, cheilion; Go', soft-tissue gonion; N', soft-tissue nasion; R7, upper cheilion region; R9, buccal region; T_4_, at the end of treatment; Sn', subnasale; ULP, upper lip point; Zy, zygomatic point

There was a moderate positive correlation between the 3D negative changes of R9 and distance of N'-Me' (rs = 0.30, *P* < 0.05) after orthodontic treatment. The correlation between negative changes of R9 and distance of Sn'-Me' was also found to be moderately positive with rs = 0.30, *P* < 0.05. Among the 3D angle measurements, only the RtCh-ULPm-LtCh angle was found to have a moderate correlation with 3D negative changes of R7 (rs = 0.31, *P* < 0.01). While in the 3D ratio items, 3D negative changes of R9 was moderately correlated with the ratio of Sn'-Me'/RtGo'-LtGo' (rs = 0.33, *P* < 0.05) and N'-Me'/RtZy-LtZy, respectively (rs = 0.30, *P* < 0.05) (Figs. 3 and 4).

The upper and lower lip protrusions of the extraction patients were more prominent than those of the non-extractive group, while other 3D facial features showed no significant differences between the two groups. Among the 3D morphological features, short distance of Sn-Me' and N'-Me', smaller ratio of Sn'-Me'/RtGo'-LtGo' and N'-Me'/RtZy-LtZy were associated with the negative change of the R9 region, implying that adult female orthodontic patients with wide and short faces may be more prone to negative changes in soft tissue during orthodontic treatment.

#### Correlation analysis of occlusal height changes and 3D facial soft tissue changes in adult females after orthodontic treatment

In the extraction group, the upper and lower anterior teeth were significantly reduced in sagittal upward tilt and protrusion after orthodontic treatment, the angle of U1-L1 increased about 14 degree while in the non-extraction group, the lower anterior teeth were increased in sagittal upward tilt and protrusion after treatment, the angle of U1-L1 decreased about 8 degree. There was no significant change in the facial vertical height before and after treatment. After orthodontic treatment, there was a weak negative correlation between the posterior height (N-Go) distance variation and 3D negative changes of R3 in facial soft tissue, indicating that an increase in the posterior height (N-Go) distance may be a risk factor for 3D negative changes in the zygomatic arch area (R3) of adult females (Additional file 1: Table S4). The orthodontist should avoid extensively using occlusal tools or techniques (such as flat bite plate, occlusal pad, etc.), so as to avoid aggravating the 3D negative changes of facial soft tissue. If it must be used, the risk of developing facial soft tissue wasting and possible “braces face” should be informed in advance.

#### Correlation analysis of adult female dental arch width changes and 3D facial soft tissue changes after orthodontic treatment

**Fig. 5:**
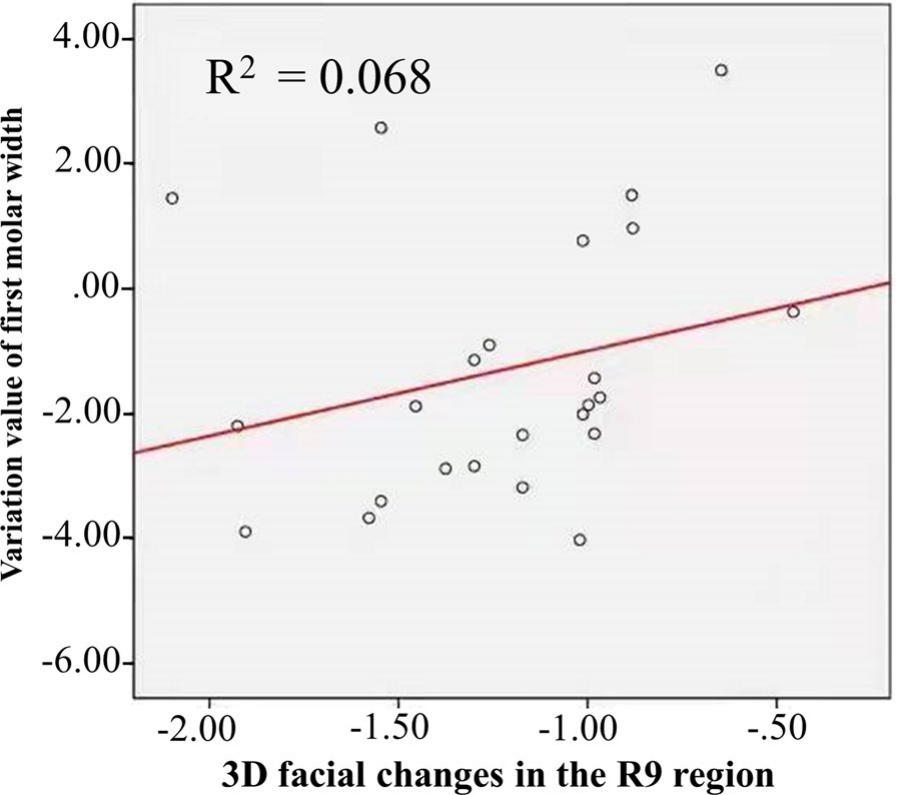
Correlation analysis between adult female dental arch width and 3D facial soft tissue changes after orthodontic treatment. R9, buccal region

Before and after orthodontic treatments, there was no statistical difference in maxillary canine width and first molar width between the extraction and non-extraction groups. There was a statistical difference in maxillary first molar width between the extraction and non-extraction groups after treatment (*P* < 0.001). A moderate positive correlation between the maxillary first molar width changes and the 3D negative change of lower cheek region (R9) in extraction group after orthodontics was found (rs = 0.41, *P* < 0.05) (Fig. 5, Additional file 1: Table S5).

## Discussion

In this pilot study, the overall protrusions of facial soft tissues in adult women were reduced by about 2 mm on average during orthodontic treatment, regardless of whether teeth were extracted or not. During orthodontic treatment, the infraorbital, cheekbone, zygomatic, subzygomatic areas as well as lower cheilion, buccal and mandibular angel in addition to temporal regions showed negative changes, with an average reduction of about 1 mm. The results showed that some facial regions like the temporal area, buccal area and cheekbone area developed negative changes in adult female facial soft tissue during orthodontic treatment, but there was no significant difference between extractive and non-extractive treatments. Before orthodontics, the upper and lower lip protrusion of patients was more prominent in the extraction group than in the non-extractive group, while other facial 3D features showed no significant difference between the two groups. After orthodontic treatment, it was found that adult female orthodontic patients with wide and short faces may be more prone to negative changes in soft tissue and an increase in posterior height (N-Go) distance may be a risk factor for 3D negative changes in the zygomatic arch area (R3). Analysis of changes in the dental arch width found a moderate positive correlation between the lower cheek region (R9) and the change in first molar width, implying the narrower the dental arch width, the more obvious the lower cheek depression.

In the present study, it was found that there was a certain trend of changes in different areas of facial soft tissue in adult patients. The tissue shrinkage changes in the face soft tissue were detected as early as 3 months after treatment but did not worsen thereafter, which was controversially different from a recent study, showing rate of facial changes was twice as fast during the first three months as that during fourth to sixth month [29].

Previously, it was believed that facial thinning in patients with tooth extraction was common, while non-tooth extraction patients generally did not exhibit negative facial changes. However, with the increase of adult patients in clinical practice in recent years, many patients without tooth extraction also have the problem of reduced facial fullness. The present study confirmed that adult women with or without tooth extraction will have negative facial trend changes because of the orthodontic process, but without significant differences in variation or the time of occurrence. When we compared the individual regions of faces, it was found that among the 11 facial regions in the extraction and non-extraction groups, only the temporal region showed statistically significant differences. Facial changes over time include changes in the facial bone and soft tissue, but studies on facial soft tissue changes over time are rare. Ashley [30] used MRI to study the thickness of female facial soft tissue at different stages and found there were significant differences in facial soft tissue thickness between a young group (21–33 years old), a middle-aged group (53–58 years old) and an elderly group (75–85 years old) in the temporal, suborbital, middle buccal and lateral buccal regions, but there was no difference in facial soft tissue thickness between the middle-aged group and the elderly group. However, the finding that the temporal area has been affected by the orthodontic treatment cannot be explained by aging since differences appeared in the present study between the beginning and end of an orthodontic treatment of 2–3 years. The temporal shrinkage of patients who had teeth extractions in the present study was about 0.3 mm more than that in those without tooth extraction and the changes of soft tissue in the temporal region were independent of the soft tissue in other parts of the face, with even the soft tissue in the temporal region with round and plump cheeks being the first to change. The reason of temporal area changes might be that during the first 3 months the fixed appliances changed the balance of the muscles attached to the temporal bone changing their position in a negative way. For subsequent changes, we need to continue to observe for a longer time after treatment.

Raskin and LaTrenta [31] found that facial fat widely exists above and below the superficial muscular-fascia system (SMAS), resulting in different facial contour shapes above these muscular-fascia systems. About 80% of subcutaneous fat is in the face and 20% in the neck. In the face, 57% of the fat is above and 43% below the SMAS. The skin on the surface of the face is connected by different ligaments to a number of key suture surface interfaces, as well as to the superficial muscular-fascial system, creating a vast network of connections. This complex network system acts as an effective mechanical shunt and amplifier, reflecting any forces transmitted within it to the entire facial system [32]. Staloff et al. [33] found that a change in facial skin is related to the movement direction of the underlying muscles and reflects their force. With the aging of skin, the force distribution of the underlying muscles is also affected and the force distribution on the muscles is compressed, with obvious spatial asymmetry. Bite force by muscle through the dermis to the epidermis can result in skin deformation. Therefore, any changes in the reticular structure that makes up the dermis can have an impact on the epidermis.

Thus, it may be speculated that bite force changes in the process of orthodontic treatments can alter the conduction force in the whole SMAS system, causing a series of cascade reaction, producing changes in the distribution of adipose tissue in the SMAS system and on the surface of the skin tissue as well as muscle itself, resulting in facial soft tissue changes. However, since few studies have explored the change of bite force during orthodontics, it is still unclear whether the change of bite force is a major cause of facial soft tissue changes. Therefore, further studies are needed. Whether aging is a risk factor for such a change is also unknown.

The first limitation of the present study is that due to the limited sample size, a sub-analysis of any difference in changes of facial soft tissues after orthodontics in different age groups could not be performed. The second limitation of this study is that the inclusion of the tooth extraction case group was not strictly differentiated according to anchorage requirements, so the assessment of lip position was inaccurate. Besides, the present study is limited since the results were obtained from a Chinese middle age sample of female patients. Therefore, the results should be extrapolated for patients with these characteristics. In addition, there is a paucity of studies on chewing efficiency and bite force changes in orthodontic patient, and these important factors were not included in the experimental design of the present study. Therefore, it remains to be investigated further whether changes in chewing efficiency and bite force are causes of facial changes in patients.

## Conclusions

Short distance of nasion to menton and subnasale to menton, long width of middle and lower face, large ratio of face height to face width are major factors leading to failure of facial aesthetics. For these patients, extraction and non-extraction of orthodontics are both easy to cause the sunken patient’s cheek and temporal areas, making the contour of face more obvious and easier to produce aesthetic risks. Changes of posterior facial height and arch width of the first molar were also risk factors for negative changes of facial soft tissues.

Teeth extraction was not a major factor for facial soft tissue changes.

## Supplementary Information

**Additional file 1:** The related factors with facial soft tissue changes.

## Data Availability

The datasets used and/or analysed during the current study are available from the corresponding author on reasonable request.

## Abbreviations

A: Subspinable
Al: Nasal ala
ANS: Anterior nasal crest
Ar: Articulare
B: Supramental
B': Soft-tissue B-point
Ch: Cheilion
En: Endocanthion
Ex: Exocanthion
FT: Fronto-temporal point
FH plane: Frankfort horizontal plane
G: Glabella
Go: Gonial
Go': Soft-tissue gonion
HP: Horizontal reference plane
Li: Labrale inferior
Ls: Labrale superior
Lt: Left
N: Nasion
Me: Menton
Me': Soft-tissue menton
N: Nasion
N': Soft-tissue nasion
Or: Orbital point
Pn: Pronasale
Po: Porion point
Pog': Soft-tissue pogonion
Rt: Right
R1: Infraorbital region
R2: Cheekbone region
R3: Zygomatic region
R4: Paranasal region
R5: Upper cheek region
R6: Sub-zygomatic region
R7: Upper cheilion region
R8: Lower cheilion region
R9: Buccal region
R10: Mandibular angle region
R11: Temporal region
SMAS: Superficial muscular-fascia system
Sn': Subnasale
Tra: Tragus
Tri: Trichion
T_0_: Before bracket bonding
T_1_: 3 Months after treatment initiation
T_2_: 6 Months after treatment initiation
T_3_: 12 Months after treatment initiation
T_4_: At the end of treatment
ULPm: Upper lip point midline
VP: Vertical reference plane
Zy: Zygomatic point
