# Supplementary material for: Profiles of facial soft tissue changes during and after orthodontic treatment in female adults
Source: BMC Oral Health. 2022 Jun 26;22:257. doi: 10.1186/s12903-022-02280-5 (PMC9233845; doi:10.1186/s12903-022-02280-5)
Supplement: Supplementary file 1 — Additional file 1: The related factors with facial soft tissue changes. [file 12903_2022_2280_MOESM1_ESM.docx]

**Profiles of Facial Soft Tissue Changes During and After Orthodontic Treatment in Female Adults**

**Running title:** Soft Tissue Changes During and After Orthodontics

Jie Gao^1,#^, Xian Wang^1,#^ , Zaixiu Qin^2,#^, Hao Zhang^1^, Donghui Guo^1^, Yuerong Xu^1,*^, Zuolin Jin^1,*^

^1^State Key Laboratory of Military Stomatology & National Clinical Research Center for Oral Diseases & Shaanxi Clinical Research Center for Oral Diseases, Department of Orthodontics, School of Stomatology, The Fourth Military Medical University, Xi’an, 710032, China

^2^Department of Stomatology, Haiyi Hospital, Zhoushan, 316000, China

^#^Jie Gao, Xian Wang and Zaixiu Qin contributed equally to this work

***Corresponding authors:**

**Zuolin Jin, MD**

State Key Laboratory of Military Stomatology & National Clinical Research Center for Oral Diseases & Shaanxi Clinical Research Center for Oral Diseases, Department of Orthodontics, School of Stomatology, The Fourth Military Medical University, No. 169 Changle West Road, Xi’an, 710032, China

Tel: +86-029-84776138

Fax: +86-29-83223047

Email: jinzuolinBM@outlook.com

**Yuerong Xu, MD**

State Key Laboratory of Military Stomatology & National Clinical Research Center for Oral Diseases & Shaanxi Clinical Research Center for Oral Diseases, Department of Orthodontics, School of Stomatology, The Fourth Military Medical University, No. 169 Changle West Road, Xi’an, 710032, China

Tel: +86-029-84776138

Email address: xyr0722@163.com

**Additional file 1**

**Image acquisition and facial soft tissue change evaluation methods**

(1) Establishment of Camper's plane: The front points of the left and right tragus, and the midpoints of the left and right nasal alar points were taken as the center. Camper's plane is the plane passing these 4 points (1). (2) The right tragus point O (0, 0, 0) was used as the rotation origin and the 2 tragus points were used to establish the axis of rotation 1. Based on the axis of rotation 1, Camper's plane was rotated 7.5 degrees upward, and the rotated plane was basically close to the true horizontal plane, which is called the reference plane 2 (**Fig. S1A**). (3) For the establishment of a transverse plane, a plane parallel to the reference plane 2 was constructed through the soft tissue nasion. (4) For establishment of the sagittal plane, a plane perpendicular to the transverse plane was made by connecting the soft tissue nasion and bilateral tragus. (5) For establishment of a coronal plane the vertical, transverse and sagittal planes were made through soft tissue nasion. (6) For the generation of a three-dimensional coordinate system, after the three-dimensional plane was established, a new three-dimensional coordinate system was generated with soft tissue nasion as the origin N '(0,0,0), where X represents the transverse direction, Y represents the vertical direction and Z represents the sagittal direction (**Fig. S1B**)


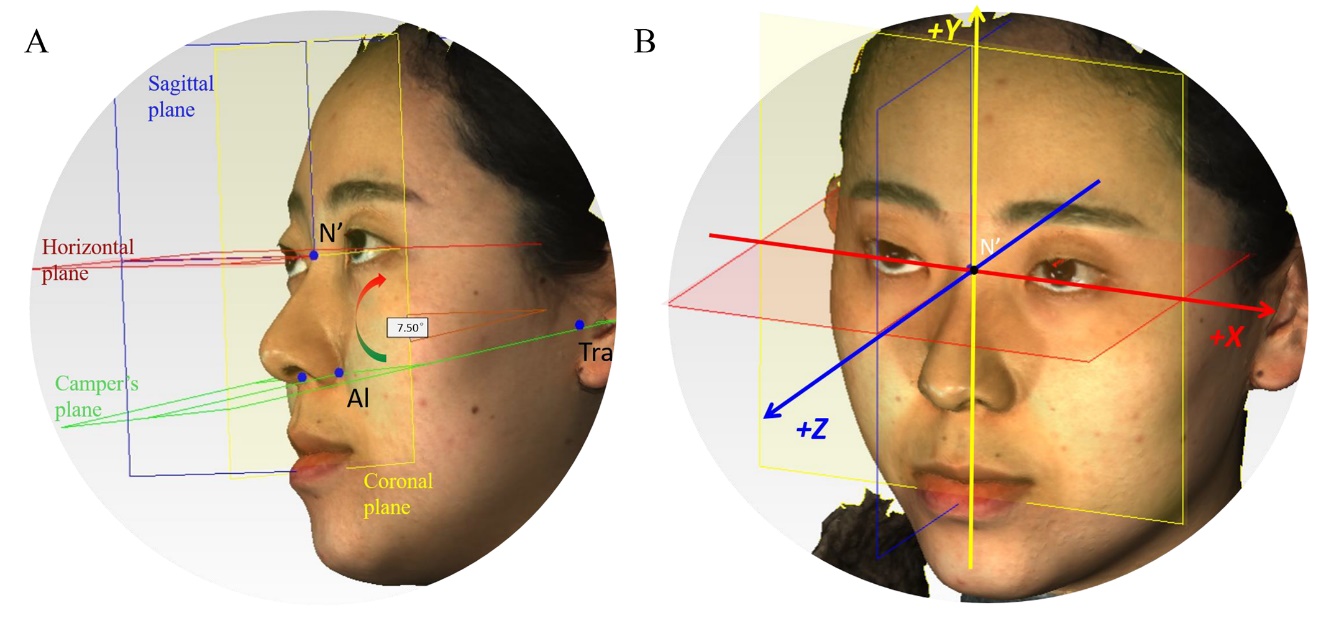


**Figure S1**. Division of the facial coordinate system and the facial area. A) establishment of the Camper's plane. B) Coronal, transverse and sagittal planes.

Abbreviations: Al, nasal ala.

(7) Images were manually registered. The T_0_ image was selected as a fixed image and T_1_-T_4_ images as floating images. (8) Reference points were selected in the frontal region and nasion region, including nasion (point 0), left and right endocanthion (points 1 and 2), and left and right exocanthion (points 2 and 3). The frontal and nasion regions of T_1_-T_4_ and T_0_ images were used as a reference to register the T_1_-T_4_ image with the T_0_ image (global registration). The images of T_1_-T_4_ and T_0_ were "accurately moved" with reference to the nasion, left and right endocanthion, and left and right exocanthion, while these points were completely overlapped. At this point, the overlapping image of T_1_-T_4_ and T_0_ with the same coordinate system could be obtained. The overlapping image with the same coordinate system was repaired to remove the fragments around the image and to retain the complete facial tissue, so as to generate the final image of facial 3D analysis in the later stage. Absolute color mapping was used for qualitative and quantitative analysis of the distance between different images. Green indicated that the soft tissue change did not exceed the allowable deviation level. Blue indicated that the soft tissue shape became flat or depressed with a negative reading. Red indicated a more convex soft tissue profile with a positive reading (**Fig. S2**). The maximum variation of 11 measurement areas on both sides and the total area were recorded.


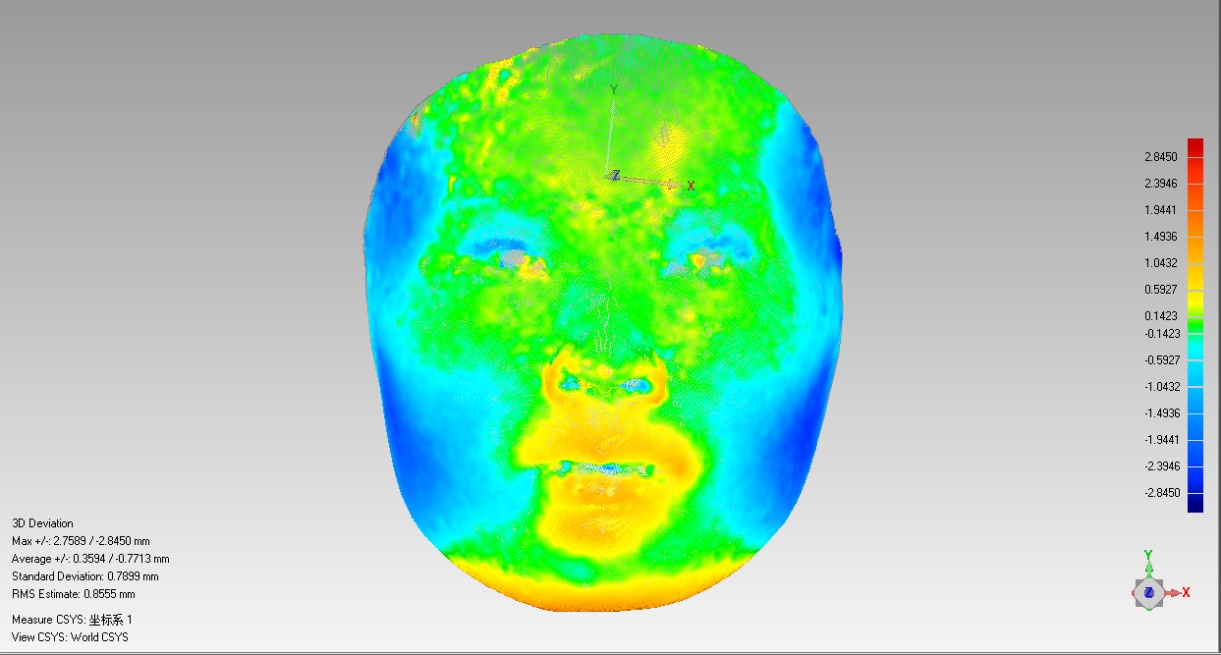


**Figure S2**. Rainbow image of facial soft tissue changes.

**Reference**

1. Ferrario VF, Sforza C, Schmitz JH, Miani A, Jr., Serrao G. A three-dimensional computerized mesh diagram analysis and its application in soft tissue facial morphometry. Am J Orthod Dentofacial Orthop. 1998;114(4):404-13.

**Additional file 1**

**Error analysis methods**

Potential experimental errors mainly included point fixation and image overlapping errors. The operator of this study fixed bilateral endocanthion, bilateral exocanthion, and nasion in T_0_ and T_1_ images of 10 subjects successively through strict marker point positioning and origin identification every other week. The positions of all the new markers and the original markers in the 3D coordinate system were calculated and a paired *t*-test was performed on the values of each fixed point using SPSS ver. 22.0 software. The results showed no statistical differences, which proved the reliability and consistency of the point fixation. For image overlapping errors, 10 patients were randomly selected, and repeated matching was performed at intervals of 1 and 2 weeks. Each time, T_1_ images were selected to overlap with T_0_ images and the Geomagic software automatically output the deviation level. The error range was 0.22 ± 0.06 mm, within which the software automatically selected the maximum area matching and the matching area was ≥ 90%.

**Table S1. Comparison of 3D line distance between extractive group and non-extractive group before orthodontic treatments**

| Measurement items (mm) | Extraction | | Non-extraction | | *P-*value |
| --- | --- | --- | --- | --- | --- |
|  | $\bar{X}\boldsymbol{\pm}$**SD** | **95% CI** | $\bar{X}\boldsymbol{\pm}$**SD** | **95% CI** |  |
| Tri-G | 56.51 ± 6.69 | 53.69, 59.34 | 55.45 ± 5.79 | 53.21, 57.70 | 0.543 |
| G-N’ | 16.25 ± 3.20 | 14.89, 17.60 | 16.81 ± 2.81 | 15.72, 17.90 | 0.502 |
| N’-Pn | 43.67 ± 3.33 | 42.26, 45.08 | 43.85 ± 3.95 | 42.32, 45.39 | 0.862 |
| Pn-Sn | 18.74 ± 1.69 | 18.03, 19.46 | 18.20 ± 1.77 | 17.51, 18.89 | 0.267 |
| Li-B’ | 11.50 ± 2.32 | 10.51, 12.48 | 11.47 ± 2.34 | 10.56, 12.38 | 0.963 |
| N’-Me’ | 119.32 ± 6.74 | 116.47,122.17 | 119.91 ± 8.83 | 116.48, 123.34 | 0.792 |
| Tri-N’ | 71.22 ± 7.06 | 68.24, 74.20 | 71.03 ± 5.99 | 68.70, 73.35 | 0.914 |
| N’-Sn | 52.47 ± 3.35 | 51.05, 53.89 | 51.95 ± 3.25 | 50.69, 53.21 | 0.573 |
| Sn-Me’ | 69.89 ± 5.83 | 67.43, 72.36 | 69.74 ± 5.78 | 67.50, 71.98 | 0.925 |
| RtTra-Go’ | 49.51 ± 6.99 | 46.56, 52.47 | 53.09 ± 7.93 | 50.01, 56.17 | 0.093 |
| LtTra-Go’ | 49.32 ± 7.03 | 46.36, 52.29 | 52.29 ± 8.63 | 48.94, 55.64 | 0.186 |
| RtGo’-Me’ | 93.93 ± 7.65 | 90.70, 97.16 | 95.12 ± 7.32 | 92.28, 97.97 | 0.569 |
| LtGo’-Me’ | 92.70 ± 7.28 | 89.63, 95.78 | 95.75 ± 7.26 | 92.93, 98.57 | 0.139 |
| RtEx-LtEx | 93.40 ± 8.12 | 89.97, 96.84 | 95.00 ± 7.96 | 91.91, 98.09 | 0.479 |
| RtTra-LtTra | 147.58 ± 8.85 | 143.84, 151.32 | 150.18 ± 7.66 | 147.21, 153.16 | 0.261 |
| RtGo’-LtGo’ | 120.53 ± 8.40 | 116.98, 124.08 | 121.96 ± 7.56 | 119.03, 124.89 | 0.521 |
| RtAl-LtAl | 38.86 ± 2.99 | 37.60, 40.13 | 39.62 ± 3.11 | 38.41, 40.83 | 0.378 |
| RtULP-LtULP | 11.70 ± 1.70 | 10.98, 12.41 | 11.47 ± 1.62 | 10.84, 12.10 | 0.627 |
| RtCh-LtCh | 49.88 ± 4.36 | 48.04, 51.72 | 49.11 ± 5.01 | 47.17, 51.06 | 0.562 |
| RtFT-LtFT | 73.6 ± 6.75 | 70.82, 76.53 | 74.80 ± 6.42 | 72.31, 77.30 | 0.540 |
| RtEn-LtEn | 37.44 ± 3.17 | 36.10, 38.78 | 38.09 ± 3.07 | 36.90, 39.28 | 0.456 |
| RtZy-LtZy | 116.29 ± 5.10 | 114.14, 118.45 | 118.36 ± 5.41 | 116.26, 120.46 | 0.165 |

Abbreviations: Al, nasal ala; B’, soft-tissue B-point; Ch, cheilion; En, endocanthion; Ex, exocanthion ; FT, fronto-temporal point; G, glabella; Go’, soft-tissue gonion; Li, labrale inferior; Ls, labrale superior; Lt, left; Me’, soft-tissue menton; N’, soft-tissue nasion; Pn, pronasale; Pog’, soft-tissue pogonion; Rt, right; Sn’, subnasale; Tra, tragus; Tri, trichion; ULPm, upper lip point midline; Zy, zygomatic point.

**Table S2. Comparison of 3D facial angle between extractive group and non-extractive group before orthodontic treatments**

| Measurement items (°) | Extraction | | Non-extraction | | *P-*value |
| --- | --- | --- | --- | --- | --- |
|  | $\bar{X}\boldsymbol{\pm}$**SD** | **95% CI** | $\bar{X}\boldsymbol{\pm}$**SD** | **95% CI** |  |
| G- Sn’-Pog’ | 16.23 ± 6.09 | 13.66, 18.64 | 16.54 ± 7.53 | 13.81, 19.35 | 0.874 |
| Pn- Sn’-Ls | 93.29 ± 14.64 | 87.02, 98.91 | 96.25 ± 9.86 | 92.54, 99.78 | 0.391 |
| Sn’-Ls⊥Li- B’ | 92.87 ± 11.97 | 87.98, 97.78 | 107.01 ± 14.53 | 101.43, 112.27 | ＜ 0.01 |
| Li- B’-Pog’ | 129.66 ± 11.84 | 125.26, 134.31 | 133.18 ± 12.18 | 128.72, 138.00 | 0.298 |
| G-N’-Pn | 143.91 ± 8.65 | 140.25, 147.56 | 145.12 ± 6.93 | 142.43, 147.81 | 0.578 |
| N’-Pn-Pog’ | 132.70 ± 8.83 | 128.96, 136.42 | 135.10 ± 4.72 | 133.26, 136.93 | 0.218 |
| N’-Pn⊥G-Pog’ | 29.39 ± 3.76 | 27.80, 30.98 | 28.74 ± 2.90 | 27.62, 29.87 | 0.489 |
| RtZy-Pn-LtZy | 118.08 ± 4.10 | 116.34, 119.81 | 116.66 ± 4.56 | 114.89, 118.43 | 0.249 |
| RtCh-ULPm-LtCh | 103.36 ± 6.69 | 100.53, 106.19 | 106.22 ± 6.83 | 103.57, 108.87 | 0.135 |
| RtGo’-Pog’-LtGo’ | 78.79 ± 3.72 | 77.22, 80.37 | 77.67 ± 4.01 | 76.11, 79.22 | 0.301 |
| RtTra-RtAl⊥RtGo’-Me’ | 24.05 ± 6.43 | 21.34, 26.77 | 22.05 ± 5.33 | 19.98, 24.11 | 0.224 |
| LtTra-LtAl⊥LtGo’-Me’ | 24.04 ± 6.69 | 21.21, 26.86 | 21.35 ± 4.71 | 19.52, 23.18 | 0.096 |

Abbreviations: Al, nasal ala; B’, soft-tissue B-point; Ch, cheilion; G, glabella; Go’, soft-tissue gonion; Li, labrale inferior; Ls, labrale superior; Lt, left; Me’, soft-tissue menton; N’, soft-tissue nasion; Pn, pronasale; Pog’, soft-tissue pogonion; Rt, right; Sn’, subnasale; Tra, tragus; ULPm, upper lip point midline; Zy, zygomatic point.

**Table S3. Comparison of 3D ratio between extractive group and non-extractive group before treatment**

| Measurement items | Extraction | | Non-extraction | | *P-*value |
| --- | --- | --- | --- | --- | --- |
|  | $\bar{X} \pm$SD | 95% CI | $\bar{X} \pm$SD | 95% CI |  |
| Tri-N’/RtGo’-LtGo’ | 0.59 ± 0.08 | 0.56, 0.62 | 0.58 ± 0.06 | 0.56, 0.61 | 0.611 |
| N’-Sn/RtGo’-LtGo’ | 0.44 ± 0.04 | 0.42, 0.46 | 0.42 ± 0.33 | 0.41, 0.44 | 0.338 |
| Sn-Me’/RtGo’-LtGo’ | 0.58 ± 0.05 | 0.56, 0.60 | 0.57 ± 0.05 | 0.55, 0.59 | 0.531 |
| N’-Me’/RtGo’-LtGo’ | 0.99 ± 0.08 | 0.96, 1.02 | 0.98 ± 0.06 | 0.95, 1.00 | 0.375 |
| N’-Me’/RtZy-LtZy | 1.02 ± 0.08 | 0.99, 1.06 | 1.00 ± 0.06 | 0.98, 1.03 | 0.237 |
| N’-Me’/RtTra-LtTra | 0.81 ± 0.06 | 0.79, 0.83 | 0.79 ± 0.03 | 0.78, 0.80 | 0.144 |
| RtEn-LtEn /RtAl-LtAl | 0.96 ± 0.09 | 0.93, 1.00 | 0.97 ± 0.12 | 0.92, 1.07 | 0.978 |
| ULPm-Li/RtCh-LtCh | 0.41 ± 0.05 | 0.38, 0.42 | 0.40 ± 0.06 | 0.37, 0.42 | 0.732 |
| Li-Me’/Go’-Me’ | 0.48 ± 0.07 | 0.45, 0.51 | 0.46 ± 0.07 | 0.43, 0.49 | 0.332 |
| Tra-Go’/N’-Me’ | 0.41 ± 0.06 | 0.39, 0.44 | 0.44 ± 0.06 | 0.42, 0.46 | 0.105 |
| N’-Sn/Sn-Me’ | 0.75 ± 0.06 | 0.72, 0.78 | 0.75 ± 0.07 | 0.72, 0.78 | 0.786 |
| Tri-Me’/RtZy-LtZy | 1.64 ± 0.13 | 1.58, 1.70 | 1.61 ± 0.09 | 1.57, 1.64 | 0.251 |
| Tri-N’/RtFT-LtFT | 0.97 ± 0.11 | 0.92, 1.02 | 0.96 ± 0.12 | 0.91, 1.00 | 0.611 |
| N’-Sn/RtZy-LtZy | 0.45 ± 0.04 | 0.44, 0.47 | 0.44 ± 0.03 | 0.43, 0.45 | 0.219 |
| RtCh-LtCh/RtEn-LtEn | 1.34 ± 0.17 | 1.27, 1.42 | 1.30 ± 0.16 | 1.23, 1.36 | 0.333 |
| RtGo’-LtGo’/RtEx-LtEx | 1.29 ± 0.07 | 1.26, 1.32 | 1.28 ± 0.85 | 1.26, 1.32 | 0.803 |

Abbreviations: Al, nasal ala; Ch, cheilion; En, endocanthion; Ex, exocanthion ; FT, fronto-temporal point; Go’, soft-tissue gonion; Li, labrale inferior; Lt, left; Me’, soft-tissue menton; N’, soft-tissue nasion; Rt, right; Sn’, subnasale; Tra, tragus; Tri, trichion; ULPm, upper lip point midline; Zy, zygomatic point.

**Table S4. Correlation between occlusal height change and 3D facial tissue change**

|  |  | w | R1 | R2 | R3 | R4 | R5 | R6 | R7 | R8 | R9 | R10 | R11 |
| --- | --- | --- | --- | --- | --- | --- | --- | --- | --- | --- | --- | --- | --- |
| N-Me  variation | Spearman correlation coefficient | .073 | .049 | .092 | .126 | -.080 | -.105 | -.119 | .136 | .029 | .136 | .077 | .062 |
|  | Significance (two sided) | .607 | .732 | .516 | .374 | .575 | .459 | .401 | .335 | .838 | .337 | .587 | .661 |
| ANS-Me  variation | Spearman correlation coefficient | .071 | -.048 | .125 | .112 | -.095 | -.086 | -.212 | .196 | .102 | .135 | .076 | -.048 |
|  | Significance (two sided) | .615 | .735 | .379 | .430 | .505 | .546 | .131 | .163 | .471 | .339 | .594 | .733 |
| N-Go  variation | Spearman correlation coefficient | -.151 | .002 | .221 | -.279 | -.074 | -.109 | -.175 | .133 | .092 | .053 | .208 | -.108 |
|  | Significance (two sided) | .286 | .991 | .116 | .045^*^ | .600 | .442 | .214 | .349 | .519 | .708 | .138 | .445 |
| Ar-Go  variation | Spearman correlation coefficient | -.015 | -.165 | .072 | -.100 | -.051 | .022 | .028 | .065 | .173 | .116 | .238 | -.050 |
|  | Significance (two sided) | .914 | .242 | .613 | .482 | .720 | .876 | .846 | .648 | .221 | .415 | .089 | .726 |

Abbreviations: ANS, anterior nasal crest; Ar, articulare; Go, gonial; N, nasion; Me, menton.

* *P* < 0.05

**Table S5. Correlation between dental arch width variation and 3D facial tissue change**

|  |  | w | R1 | R2 | R3 | R4 | R5 | R6 | R7 | R8 | R9 | R10 | R11 |
| --- | --- | --- | --- | --- | --- | --- | --- | --- | --- | --- | --- | --- | --- |
| Distance variation between the cusp points of left and right maxillary canine | Spearman correlation coefficient | -.071 | .068 | -.094 | -.222 | .035 | -.031 | -.245 | -.264 | -.242 | -.081 | -.104 | -.118 |
|  | Significance (two sided) | .741 | .752 | .663 | .297 | .872 | .880 | .249 | .236 | .254 | .706 | .628 | .397 |
| Distance variation between central fossa of left and right first molars | Spearman correlation coefficient | .153 | .325 | .249 | -.236 | -.074 | .187 | -.219 | .236 | .399 | . 407 | -.225 | .375 |
|  | Significance (two sided) | .476 | .121 | .163 | .267 | .730 | .185 | .305 | .267 | .054 | .048^*^ | .834 | .071 |

* *P* < 0.05.
